# Interior modification of *Macrobrachium rosenbergii* nodavirus-like particle enhances encapsulation of VP37-dsRNA against shrimp white spot syndrome infection

**DOI:** 10.1186/s12917-024-03936-w

**Published:** 2024-03-08

**Authors:** Itsares Muikham, Orawan Thongsum, Somkid Jaranathummakul, Atthaboon Wathammawut, Charoonroj Chotwiwatthanakun, Pitchanee Jariyapong, Wattana Weerachatyanukul

**Affiliations:** 1 Department of Anatomy, Faculty of Science, Mahidol University, Rama 6 Rd., Rachathewi, Bangkok, 10400 Thailand; 2 Department of Anatomy, Faculty of Medicine, Srinakharinwirot University, Bangkok, Thailand; 3 Faculty of Science, Mahidol University, Nakhonsawan Campus, Nakhonsawan, Thailand; 4 Center of Excellence for Shrimp Molecular Biology and Biotechnology (Centex Shrimp), Mahidol University, Bangkok, Thailand; 5 Department of Medical Science, School of Medicine, Walailak University, Thasala District, Nakhonsrithammarat, 80160 Thailand

**Keywords:** MrN Virus-like particle, WSSV, dsRNA, VP37

## Abstract

**Background:**

Application of a virus-like particle (VLP) as a nanocontainer to encapsulate double stranded (ds)RNA to control viral infection in shrimp aquaculture has been extensively reported. In this study, we aimed at improving VLP’s encapsulation efficiency which should lead to a superior fighting weapon with disastrous viruses.

**Results:**

We constructed 2 variants of chimeric *Macrobrachium rosenbergii* nodavirus (MrNV)-like particles (V1- and V2-MrN-VLPs) and tested their efficiency to encapsulate VP37 double stranded RNA as well as WSSV protection in *P. vannamei.* Two types of short peptides, RNA-binding domain (RBD) and deca-arginine (10R) were successfully engineered into the interior surface of VLP, the site where the contact with VP37-dsRNA occurs. TEM and dynamic light scattering (DLS) analyses revealed that the chimeric VLPs remained their assembling property to be an icosahedral symmetric particle with a diameter of about 30 nm, similar to the original MrN-VLP particle. The superior encapsulation efficiency of VP37-dsRNA into V2-MrN-VLP was achieved, which was slightly better than that of V1-MrN-VLP but far better (1.4-fold) than its parental V0-MrN-VLP which the mole ratio of 7.5–10.5 for all VLP variants. The protection effect against challenging WSSV (as gauged from the level of VP37 gene and the remaining viral copy number in shrimp) was significantly improved in both V1- and V2-MrN-VLP compared with an original V0-MrN-VLP template.

**Conclusion:**

MrN-VLP (V0-) were re-engineered interiorly with RBD (V1-) and 10R (V2-) peptides which had an improved VP37-dsRNA encapsulation capability. The protection effect against WSSV infection through shrimp administration with dsRNA + V1-/V2-MrN VLPs was experimentally evident.

**Supplementary Information:**

The online version contains supplementary material available at 10.1186/s12917-024-03936-w.

## Introduction

Virus like particles (VLP) are protein-based nanoparticles that are versatile for many applications, especially for delivery system [1] and for vaccine development to prevent harmful viruses [2]. In shrimp aquaculture, two VLPs derived from *Macrobrachium rosenbergii* nodavirus (MrNV) and hypodermal and hematopoietic necrosis virus (IHHNV) have been used as nano-containers to deliver dsRNA to knockdown important viral protein genes, VP28 and VP37, of white spot syndrome virus (WSSV) [3–5]. They also function as immunostimulant to trigger innate immune system both cellular and humeral responses [6, 7]. Although, the superior viral protection effect of the encapsulated dsRNA in MrN-VLPs over the naked dsRNA was observed, however, the remaining viral copy number in shrimp tissues leading to a gradual drop-death of the viral infected shrimp at the end of experiment has posted the challenging issue to be solved. One of the most comprehensive explanations for this phenomenon is due to the limitation of approximately 40–50% of the dsRNA [8] being loaded into VLP’s cavity leading to the non-satisfactory effect of the therapeutic dsRNA in viral elimination. Improvement of cargo loading through VLP modification to enhance the protection effect of encapsulated VLP against WSSV infection is thus the major aim of this study.

Modifications of VLPs both exteriorly and interiorly through an engineering of the viral capsid have been extensively reported to promote VLP functionalization, such as altering their tropism to enhance a cellular uptake pathway [9] or programmable cargo loading [10] with very minor changes in their capsid structures [11]. One of the most extensively modified VLPs that has been used for encapsulation purposes is the cowpea chlorotic mottle virus (CCMV) [12] due to its simple VLP structure and self-reassembling control [13]. Since the *N*-terminus of the CCMV capsid protein is projected inwards into VLP’s cavity, modification of this region has been investigated extensively for an enhanced cargo encapsulation using multiple strategies such as electrostatic re-enforcement or chemical conjugation employing a biorthogonal handle [14, 15]. As its close similarity with CCMV, MrN-VLP possesses a positively charged amino acids at the *N*-terminus facing into the capsid interior with its high affinity towards negatively charged nucleic acid interaction [3, 16]. It is thus reasonable to propose that genetic engineering of MrNV capsid interior via a placement of positively charged amino acids or the known RNA binding motif should thus directly enhance cargo encapsulation of MrN-VLP. The versatility of MrN-VLP for genetic modification has also extended to its *C*-terminus (projecting exteriorly to form the 4-bladed protruding structure for receptor binding) [17, 18] to functionalize its specific binding towards target cells. In this regard, we have decorated the protruding (P) domain of MrN-VLP with GE11 peptide to enhance specific binding and internalization towards epidermal growth factor receptor (EGFR) positive colorectal cancer cells, SW480 [19, 20]. Together with many superior physical properties of MrN-VLP beyond other biological containers, particularly its tolerance to harsh conditions and enzymatic digestion, it stands a suitable candidate to be developed as a biological weapon to fight against viral infection in shrimp and other aquatic animals.

## Materials and methods

### Designs of interiorly modified V1- and V2‑MrN-VLP variants

**Fig. 1:**
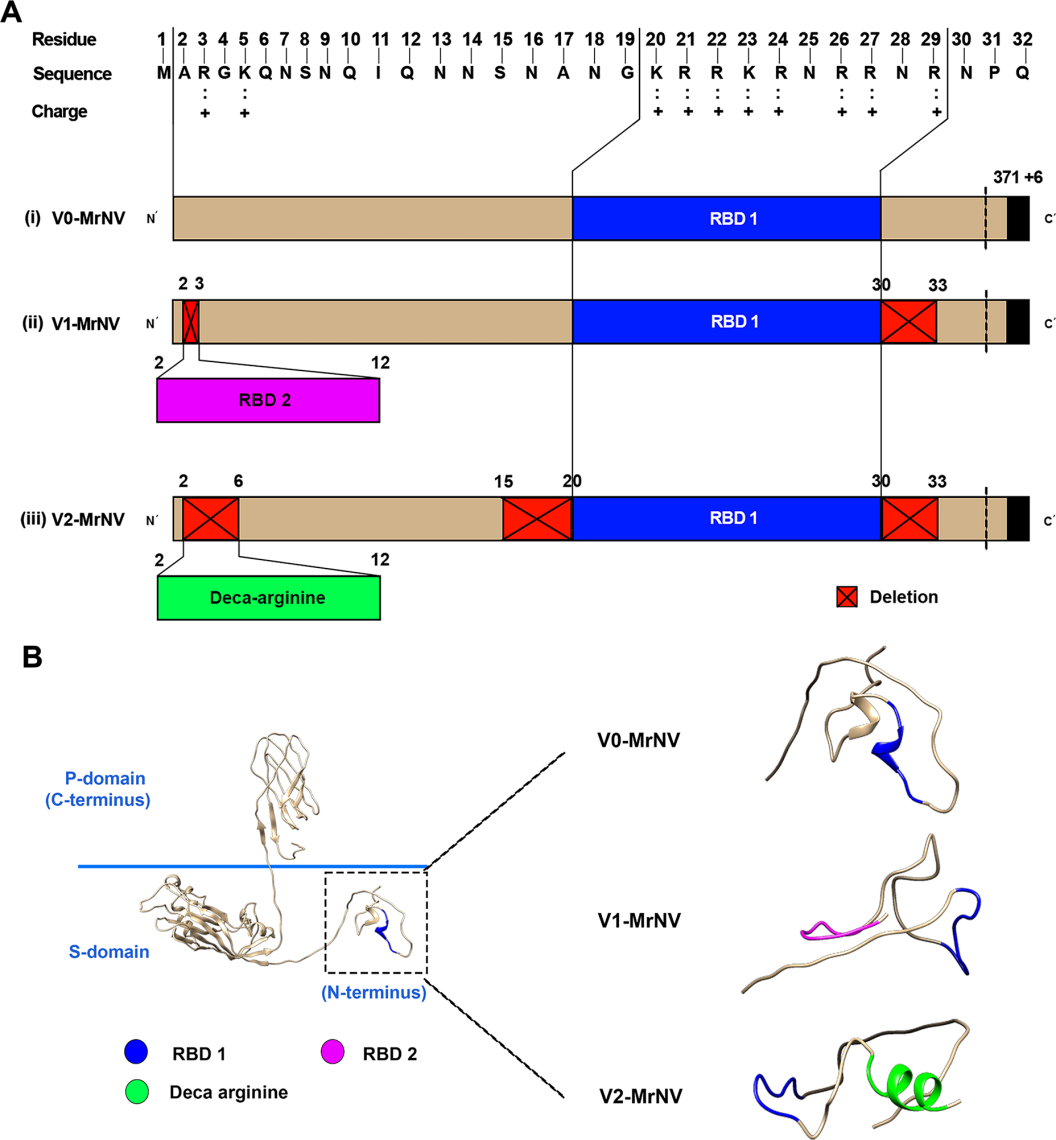
Schematic diagram of MrN-VLP constructs and deduced three dimensional structures of the modified *N*-termini of VLPs. A: linear stretches of MrNV capsid protein containing an inherent RNA-binding domain (RBD1, blue) at its *N*-terminus and the extended hexa-histidine residues at the end of *C*-terminus. Red boxed areas with crosses indicate deleted amino acids in which foremost area is replaced by the additional RNA-binding domain (RBD2, pink) or deca-arginine peptide (10R, green). B: predicted 3D ribbon models of chimeric MrN-VLPs containing capsid’s exterior, shell and capsid’s interior (left), the latter of which contains *N*-terminus amino acids which are modified by RBD (V1-MrNV) or 10R (V2-MrNV) (right)

The peptides of repeated RNA binding domain (RBD: KRRKRNRRNR to construct V1-MrN-VLP) [16] and deca-arginine (10 A: RRRRRRRRRR to construct V2-MrN-VLP) were placed into the sequence of normal (V0) capsid MrNV protein (available in National Center for Biotechnology Information (GenBank accession # EU150129). Peptide placement was performed through both deletion and insertion of the desirable peptides as depicted in Fig. 1. In V1 variant, the RBD peptide was inserted to replace a single MrNV amino acid at positions 2–3, while deleting some amino acids at positions 30–33, rendering doubled RBDs in V1 construct. In V2 variant, the MrNV amino acids at positions 2–6, 15–20 and 30–33 were deleted and replaced by a deca-arginine (10R) peptide at the first deletion site, rendering RBD + 10R feature in V2 construct (see Fig. 1A for details). Three-dimensional (3D) homology-based models of all interiorly modified V1 and V2 chimeric MrN-VLPs were created by the Protein Homology/analogy and Recognition Engine version 2.0 (Phyre2) protein folding prediction server (http://www.sbg.bio.ic.ac.uk/~phyre2/html/page.cgi) in comparison with the original V0-MrN-VLP template (PDB#6H2B) following the described protocol [19]. The icosahedral models of the chimeric MrN-VLPs were created based on a referencing atomic structure of T = 3 quasi-equivalent icosahedral V0-MrN-VLP structure [18]. All visualization was performed using the UCSF Chimera software (https://www.cgl.ucsf.edu/chimera).

### Expression and purification of recombinant V1- and V2‑MrNV capsid proteins

Expression and purification of V1- and V2‑MrNV capsid proteins were carried out following previously described protocol [19]. Briefly, V1- and V2-MrNV capsid sequences were commercially constructed into the pET16b expression vector (General Biosystems, Durham, NC) and transformed into the competent *E. coli* (BL21), respectively. The positive clones were further inoculated in LB broth containing 50 µg/ml of ampicillin overnight at 37 °C until the absorbance of 0.6–0.8 at 600 nm (A600) was reached. Protein expression was induced by 1 mM IPTG at 16 h, 25 °C for overnight and the cells were pelleted by centrifugation (5400×g, 10 min, 4 °C). The re-suspended cells were ruptured by sonication on ice (100 W, 10 s, 5 cycles), centrifuged (12,000×g, 10 min) to discard any cellular debris. The collected supernatant was loaded into a nickel affinity chromatographic column which was then washed and eluted by multi-steps of imidazole-based solutions. Protein fractions were collected and analyzed by SDS-PAGE and further verified by immunoblotting. The concentrations of purified recombinant V1- and V2‑MrNV proteins were measured by a NanoDrop 2000 Spectrophotometer (Thermo Fisher Scientific, Delaware, CA).

### Characterization of V1- and V2‑MrN-VLPs

To investigate whether chimeric V1- and V2-MrN-VLPs retained their self-assembling property to form icosahedral particles, negative staining transmission electron microscopy (TEM) was performed. Approximately 10 µl of purified VLPs was pipetted onto the carbon-coated EM grids (Electron Microscopy Sciences, Hatfield, PA) and allowed to stand for 30 s. Non-adhering VLPs were washed away by Milli-Q water and blotted away by a filtered paper. To the adhered VLPs, 2% uranyl acetate was added and allowed to stand for 30 s and repeated the washing and blotting away steps as mentioned above. Thereafter, the samples were viewed under a JEOL1230 transmission electron microscope operated at 120 kV (JEOL, Tokyo, Japan).

Dynamic light scattering (DLS) was used to determine the size of particles [21] in all types of VLP variants. The particles of MrN‑VLPs were resuspended in PBS, diluted to make a final concentration of 1 mg/mL and further dispersed by 0.45-µm filter (Millipore, Darmstadt, Germany). A suspension of 1 mL was then pipetted into a Zetasizer cells DTS0012 (Malvern Panalytical, Malvern, United Kingdom) and the Z-average diameter from three independent experiments were calculated and expressed as mean ± S.D.

### Production of VP37 dsRNA

VP37 dsRNA was synthesized following our protocol published previously [5]. Briefly, the VP37 gene was amplified using specific primers incorporating T7 promoter (Supplementary Table 1) and subjected to in vitro transcription using the T7 RiboMAX™ Express Large Scale RNA Production System (Promega, Madison, WI). The dsRNA concentration and quality were measured and checked by a NanoDrop 2000 spectrophotometer (Thermo Fisher Scientific, Wilmington, DE) and 1% agarose gel electrophoresis before its application.

### Encapsulation efficiency of V1- and V2-MrN-VLPs

The encapsulation efficiency of a nucleotide-based substance, VP37 double stranded RNA (VP37dsRNA), into V1- and V2-MrN-VLPs was investigated in comparison with V0-MrN-VLP following the described protocol [5]. Briefly, 10 µg of the purified VLPs were treated with disassembling buffer containing 1 mM ethylene glycol tetra-acetic acid (EGTA) and 20 mM dithiothreitol (DTT). An equal amount of VP37dsRNA was added into the solution of a disassembled VLP (1 h, 4 °C). Later, 100 mM CaCl_2_ was slowly added to the mixture to reach a final concentration of 5 mM. The mixture was loaded into a 100 kDa cut-off Centricon filtration device (Millipore, Darmstadt, Germany) and then subjected to centrifugation (2,000×g, 4 °C). The retentate on the filtration membrane containing VLP suspension was collected whereas the free dsRNA and excessive solution were discarded into the flow-through. The VP37 dsRNA loaded VLPs were heated at 95 °C in SDS-PAGE loading dye for breaking down all tightly assembled VLPs and used for gel electrophoresis.

To calculate the encapsulation efficiency, the band intensities of the loaded VP37 dsRNA and MrNV proteins were quantified densitometrically using an ImageJ software (NIH, Bethesda, MA, USA) against the standard curves of known amounts of the respective dsRNA and MrNV protein (see supplementary Fig. 1). Thereafter, the percentages of loaded VP37 dsRNA into VLP variants were quantified in comparison with the starting amount (10 µg) of VP37 dsRNA that was used in each encapsulation experiment. This encapsulated amount of VP37 dsRNA was converted into mole number based on the molecular weight of a single unit of dsRNA (540 kDa) and MrN-VLP subunit (single capsomer = 43 kDa). Based on the previous report, a full particle of MrNV is T = 3 equivalence, which consists of 180 capsomers, rendering the total molecular weight of 7740 kDa. Finally, the obtaining mole number of encapsulated dsRNA and the mole number of MrN-VLP proteins (full particle) was calculated and compared between chimeric VLP and V0-VLP.

### Solid phase binding assay

Comparative binding efficiency of chimeric MrNVs and the parental VLP was determined by a solid phase binding assay (modified ELISA) [22]. All types of MrN-VLPs were serially diluted in the range of 0.125 to 2 mg/mL (5 serial dilutions) and subjected to particle disassembly by a disassembling buffer described above (room temperature, 1 h). To the nitrocellulose membrane, 1 µL of each disassembled VLP solutions were blotted onto the membrane and allowed to completely dry on the membrane. The non-specific binding was blocked with 5% BSA in PBS containing 0.01% Tween-20 (0.01% PBST) for 1 h at room temperature. Disassembled VLPs were incubated with digoxigenin (DIG) conjugated VP37 dsRNA (1 h, 4 °C), washed extensively and followed by anti-DIG-alkaline phosphatase (1:500) for 2 h at 4 °C. After washing, the membrane was developed by BCIP/NBT color development substrate (5-bromo-4-chloro-3-indolyl-phosphate/nitro blue tetrazolium; Promega, Wisconsin, USA) overnight at 4 °C. The reaction was stopped by rinsing with PBS and the intensity of each immunoreactive band was recorded and quantified by an ImageJ software (https://imagej.nih.gov/ij/index.html). The data were expressed as mean ± S.D. calculated from the triplicated experiments.

#### WSSV challenge and shrimp treatment with VP37 dsRNA encapsulated chimeric VLPs

The comparative protection ability of chimeric versus normal VLPs encapsulating VP37 dsRNA against white spot syndrome virus was examined in white pacific shrimp, *Penaeus vannamei.* The animal handling protocol was approved by the Animal Ethics Committee, Walailak University (Protocol No. 020/2019). A total 90 specific pathogens free (SPF), juvenile shrimp (5 ± 0.8 g body weight) were obtained from quarantine facility at the Center of Excellence for Aquaculture Technology and Innovation, Walailak University. They were equally divided into 5 groups to receive an intramuscular injection (IM) with either PBS (control) or 5 µg of empty V0-MrN-VLP or its variants (V1- or V2-MrN-VLPs) (1 µg/g shrimp) that encapsulated VP37 dsRNA (at variable concentrations due to their different encapsulation efficiency). After 24 h of injection, all groups were secondarily IM injected with a 10^− 6^ diluted WSSV stock titrated to cause 100% shrimp mortality by 4 days post-infection (p.i.) and their tissues were collected for further analysis.

### Expression level of VP37 gene and WSSV copy number

Gill tissues from 5 individual shrimp were collected at 12, 24 and 48 p.i for determining the VP37 gene expression and WSSV copy number using specific sets of primers (Supplementary Table 1) following our published protocol [3–5]. Briefly, Either DNA or total RNA from gills of infected shrimp were extracted and reversely transcribed into cDNA template. The PCR amplification condition consisted of a holding step at 95 °C for 115 s; 40 cycles of denaturation at 94 °C for 30 s, annealing at 55 °C for 30 s, and elongation at 72 °C for 30 s; and a final elongation step at 72 °C for 5 min. The relative expressions of the VP37 genes were determined using the 2^−ΔΔCt^ method in comparison with the internal control, elongation factor-1 (EF-1α). WSSV copy number was quantitated against a standard curve of a serially diluted pGEM-T easy vectors. The data were expressed as mean ± SE of five replicates and statistically compared using a two-way ANOVA with Turkey’s multiple comparisons. The statistical analyses were performed using SPSS software (Version 17.0) and GraphPad Prism software. Statistical differences were considered at *P* < 0.05.

## Results

### Structures of MrN‑VLP variants

The putative structures of V1- and V2-MrN-VLPs were constructed based on the structure of V0-MrN-VLP parental template (PDB# 6H2B). Their comparison was performed using Phyre2 algorithm and Chimera software. Construction of VLP variants was based on replacement of short peptides and deletion of some amino acids from the original MrNV peptide chain as demonstrated by the linear, two-dimensional (2D) alignment in Fig. 1A. Ten amino acids of either RNA-binding domain (RBD) were inserted into the positions 2–3 (V1-) or deca-arginine peptide (10R) into the positions 2–6 (V2-), whereas the amino acids positioned 30–33 (V1- and V2-) and 15–20 (V2-) were deleted from the original MrNV template. The predicted 3D ribbon models of V1-MrN-VLP in Fig. 1B demonstrated the presence of an inserted RBD domain (RBD2 at positions 2–10, pink) in addition to the original RBD peptide (RBD1 at positions 20–29, blue). Similarly, the modified N-terminus of V2-MrN-VLP contained the originally located RDB1 domain (blue) and the apparently protruding 10R peptide (green) in the VLP’s interior (Fig. 1B).

### Protein profiling and characterization of V1- and V2- MrN-VLPs

**Fig. 2:**
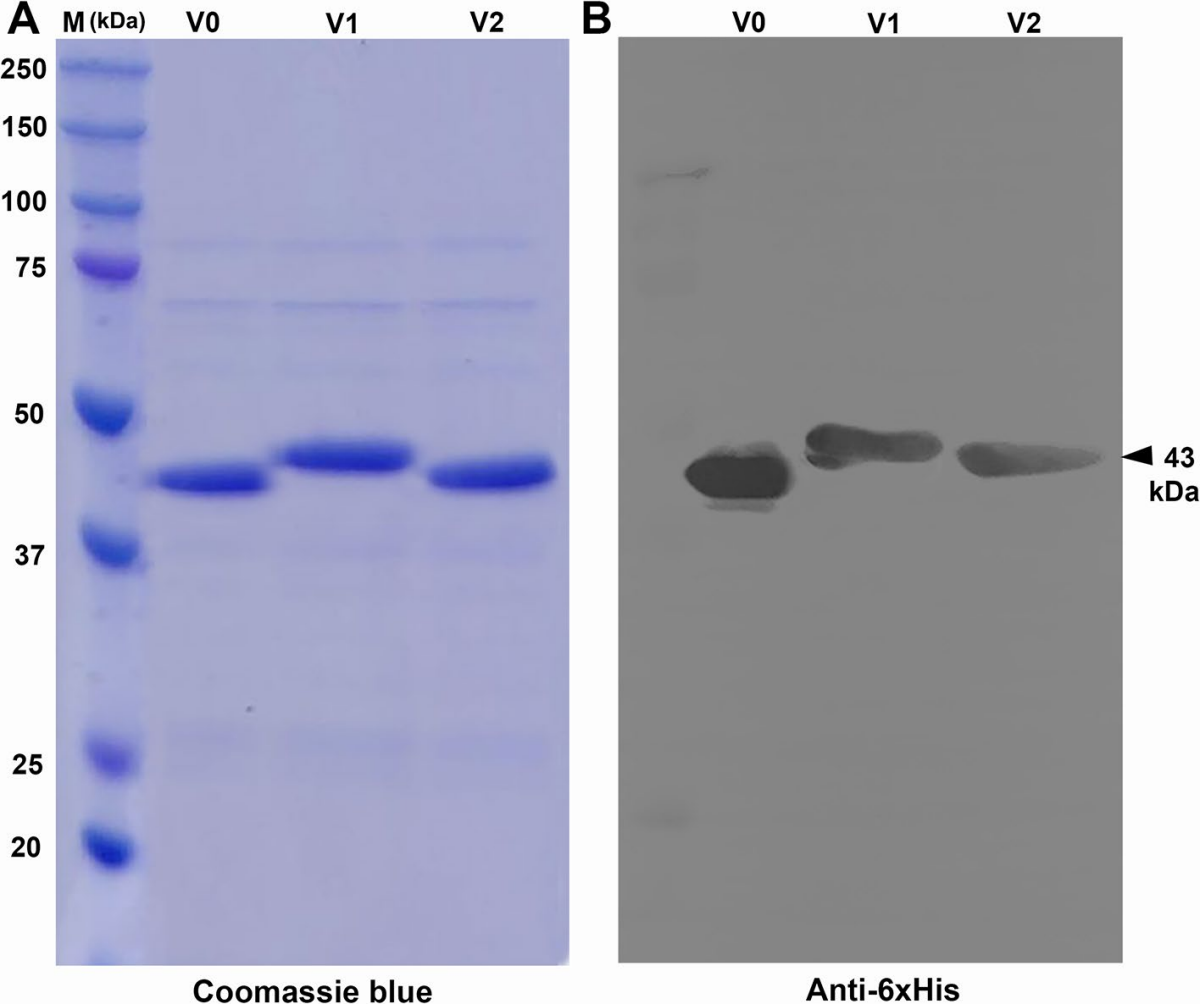
Protein profiles of chimeric MrN-VLPs and Western blotting. Purified recombinant V0- V1- and V2-MrN-VLPs were resolved by 12.5% SDS-PAGE and stained with Coomassie blue (**A**), transferred to nitrocellulose membrane and further stained with anti-His antibody (**B**). Note a single purified protein band of MrNV capsid protein at 43 kDa (arrowhead)

**Fig. 3:**
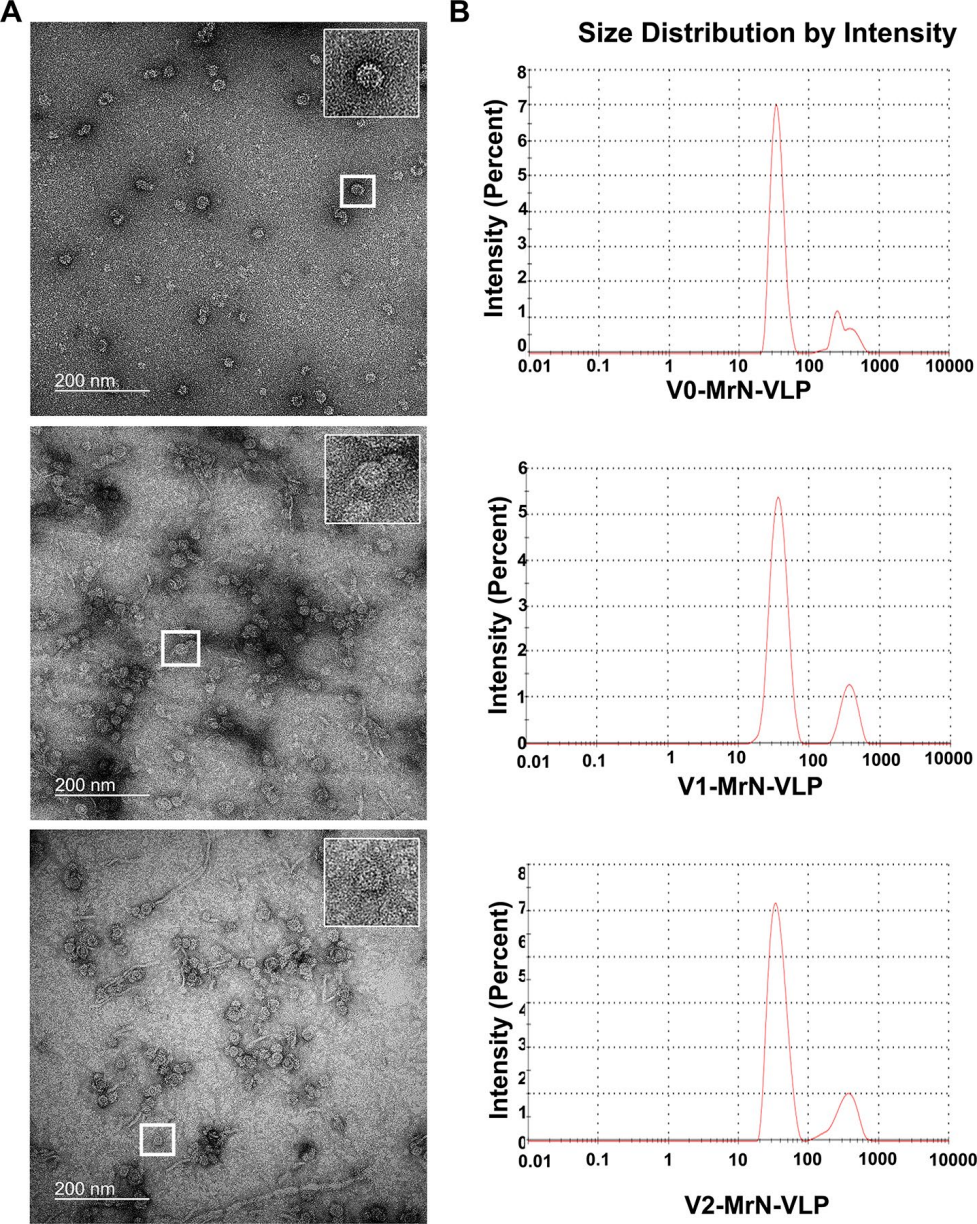
Structural analysis of MrN-VLP variants. **A**: low magnification of negative transmission microscopy (TEM) of V0-, V1- and V2-MrN-VLPs (panel A) with a unique mulberry-like icosahedral feature (insets). **B**: the averaged particle size plots analyzed by a dynamic light scattering (DLS) of all three VLP particles

Coomassie blue staining profiles of recombinant V1- and V2-MrNV capsid proteins showed a single protein band with a molecular mass of about 43.0 kDa (Fig. 2A). A slightly higher molecular mass of V1-MrN-VLP of about 43.5 kDa was also notable. This protein band well-reacted with anti-His antibodies, recognizing hexa-histidine residues which were conjugated to the C-termini of all VLP subtypes (Fig. 2B). Structural analysis was performed to confirm whether chimeric VLPs could self-assemble to form the VLP structure using a negative TEM microscopy. It was found that both V1- and V2-variants formed an icosahedral mulberry-like VLP structure with diameter approximate 30 nm (Fig. 3A) similar to that of the original V0-MrN-VLP previously reported [8].

We further quantified hydrodynamic diameter of chimeric VLPs using dynamic light scattering (DLS) analysis. The dispersing diameters of V1- and V2- MrN-VLPs were in the range of 35–40 nm where their average particle diameters were calculated to be 37.60 ± 9.98 nm and 36.89 ± 10.18 nm, respectively (Fig. 3B), which was well-correlated with the diameters obtained from TEM measurement. These data confirmed that placement of either RBD or 10R short peptide in the *N*-terminus of capsid protein did not affect the VLP structural formation and its overall diameter remained unchanged.

### Encapsulation ratio of the dsRNA into VLPs and their binding efficiency

**Fig. 4:**
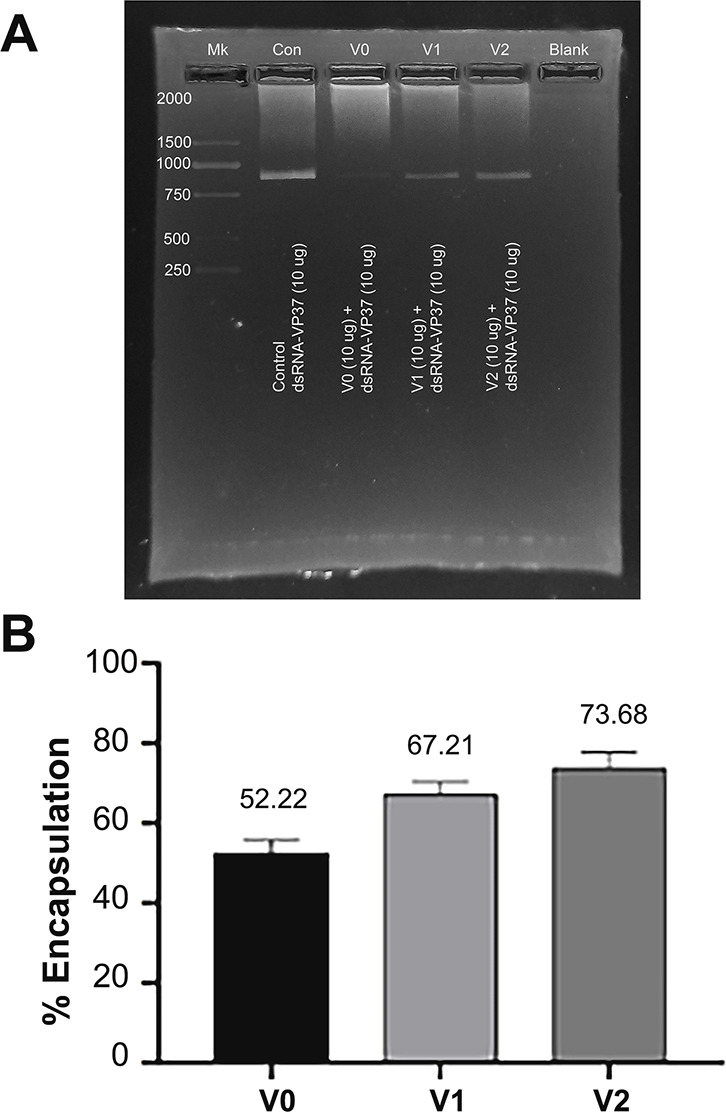
Profiles and densitometric analysis of dsRNA encapsulated into chimeric MrN-VLP variants. Ten micrograms of dsRNA (843 bp) were encapsulated into an equal amount of V0-, V1- and V2-MrN-VLPs (**A**) and their band intensity was analyzed densitometrically by ImageJ software (**B**). The percentages of dsRNA encapsulation were calculated against the standard curve (3–15 µg) of dsRNA from the triplicated experiments and shown on the top of each VLP bar

We performed a triplicated densitometric analysis to check the amount of VP37-dsRNA encapsulated into the cavities of chimeric VLPs and further calculated the mole ratio of the loaded VP37-dsRNA into the VLPs. Calculation of the amount of dsRNA after being loaded into VLPs was performed by densitometric analysis of dsRNA band’s intensity resolved in agarose gel (Fig. 4A) against dsRNA standard curve (ranging from 3 to 15 µg, Supplementary Fig. 1). Starting from 10 µg dsRNA, we found that the percentages of dsRNA encapsulated into each type of VLP were about 52.22 ± 3.57%, 67.21 ± 3.18% and 73.68 ± 4.09% (or 5.22, 6.72 and 7.37 µg) for V0-, V1- and V2-variants, respectively (Fig. 4B; Table 1). The highest mole ratios between encapsulated dsRNA (540 kDa) and VLP (7,740 kDa) was found in V2-MrN-VLP which was 10.55 ± 0.59 (Table 1). The mole ratios of V1- and V0-MrN-VLP were 9.63 ± 0.46 and 7.48 ± 0.51, which were about 1.09- and 1.4-fold less than that of V2-MrN-VLP. Statistical analysis showed significant differences (*P* < 0.05) in dsRNA loading between chimeric VLPs (V1 and V2) in comparison to V0-VLP, but no difference was found between V1- and V2 variants.

**Fig. 5:**
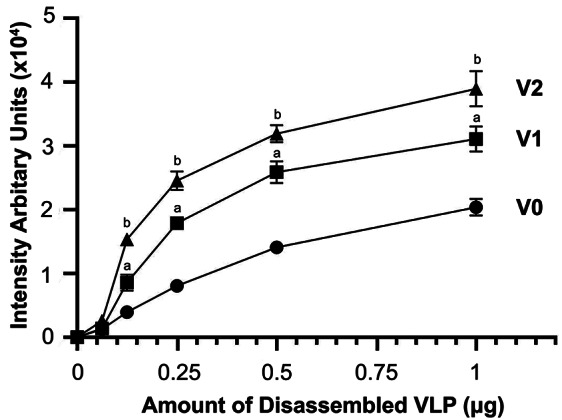
Binding efficiency of dsRNA and three subtypes of MrN-VLPs using a solid phase binding assay. The data were collected and analyzed from triplicated experiments and expressed as mean ± S.D. Letters a and b denote statistical difference compared between the triplicated data point of V1-V0 and V2-V0 groups

We further performed solid phase binding assay to determine the binding efficiency between VP37 dsRNA and VLPs. The binding curve in Fig. 5 revealed that V2-MrN-VLP always had the best binding efficiency with dsRNA at all amounts of VLP used. At 0.25 µg/ml VLP, for instance, the arbitrary intensity values of 0.82 × 10^4^, 1.75 × 10^4^ and 2.43 × 10^4^ units were obtained for V1-, V1 and V2-variants. Similar trend of results was noted in all points of data, indicating that V2-MrN-VLP possesses the best binding affinity towards dsRNA cargo among all VLPs studied.

**Table 1:** Encapsulation efficiency and mole ratio of VP37-dsRNA with the three variants of MrN-VLPs

Type of VLP	dsRNA encapsulation			
	Amount of dsRNA (µg)^a^	Percent encapsulation^b^	Average mole number	Mole ratio (dsRNA/VLP)
V0- MrN-VLP	5.22 ± 3.57	52.22	0.97 × 10^− 12^	7.48 ± 0.51
V1-MrN-VLP	6.72 ± 3.18^c^	67.21	1.24 × 10^− 12^	9.63 ± 0.46
V2 MrN-VLP	7.37 ± 4.09^c^	73.68	1.36 × 10^− 12^	10.55 ± 0.59

^a^Determined by densitometric analysis of dsRNA’s band intensity in a agarose gel electrophoresis against the standard curve of a known amount of dsRNA (from 3–15 µg)

^b^Percent encapsulation = the estimated amount of dsRNA/10 µg×100, where 10 µg is the starting amount of dsRNA used in encapsulation experiments

Letter c = statistical differences between the V0, V1 and V2 groups

### Effective silencing of VP37 gene and WSSV copy number by VP37dsRNA encapsulated chimeric VLPs

**Fig. 6:**
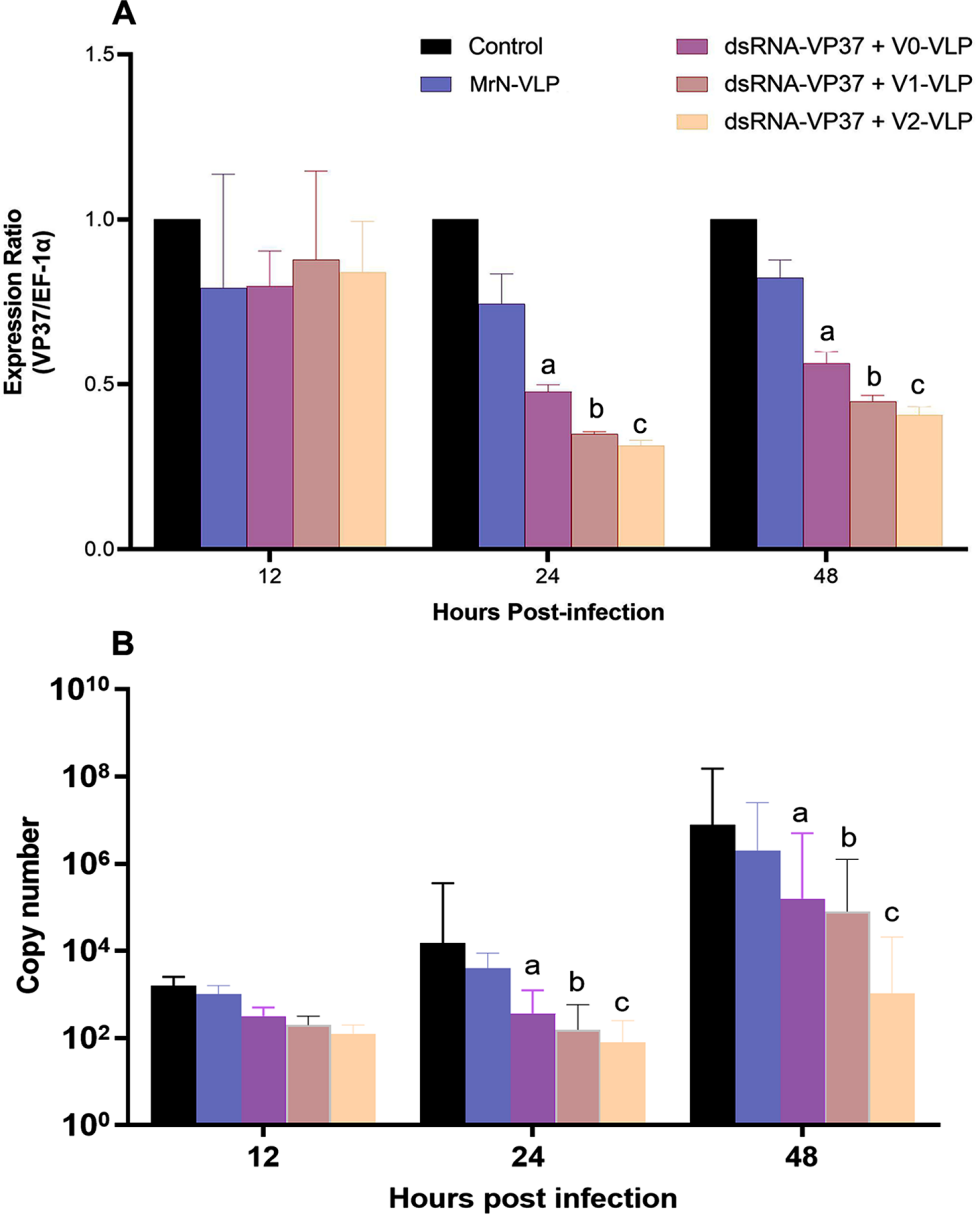
The level of VP37 gene silencing (**A**) and WSSV copy number (**B**) by MrN-VLP and encapsulated dsRNA into three types of VLPs. Letters on the bars represent significant differences between MrN-VLP and dsRNA + V0-VLP (**a**), dsRNA + V1-VLP and dsRNA + V0-VLP (**b**) and dsRNA + V2-VLP and dsRNA + V0-VLP (**c**)

The comparative protection efficiency of chimeric MrN-VLP that carried VP37 dsRNA against WSSV was also tested in *P. vannamei* challenged with the inoculum of WSSV. We first performed an initial comparison between the protection efficiency between an encapsulated VP37 dsRNA (in the V0-MrN-VLP) and empty MrN-VLP. Upon administration of VP37-dsRNA + V0-VLP into the shrimp and followed by WSSV challenge, a significant reduction (*P* < 0.05) of VP37 transcripts (analyzed by qRT-PCR) in shrimp was clearly observed compared with those receiving PBS (vehicle control) and empty MrN-VLP (Fig. 6A), consistent with our study reported earlier [5]. Interestingly, the more pronounced silencing efficiency of VP37 genes was further evident in shrimp treated with VP37 dsRNA + V1-VLP or VP37 dsRNA + V2-VLP, which was significantly different (*P* < 0.05) from the dsRNA encapsulated V0-VLP counterpart, either at 24 or 48 h post-WSSV challenge (Fig. 6A). There was no significant difference of VP37 gene levels observed between shrimp receiving VP37 dsRNA + V1-VLP or VP37 dsRNA + V2-VLP, suggesting the high efficiency of both chimeric VLP variants that may be used alternatively in the nucleotide-based delivery applications.

In conjunction with the suppression of VP37 gene, a significant lower in WSSV copy number was observed in shrimp injected with either VP37 dsRNA + V1-VLP or VP37 dsRNA + V2-VLP. WSSV copy number in control and MrN-VLP groups increased dramatically from 24 to 48 h. post challenge (Fig. 6B). At 48 h p.i, shrimp injected with PBS (control) had a viral copy number of 5.06 × 10^6^, while those injected with VP37 dsRNA + V1-VLP or VP37 dsRNA + V2-VLPs showed the viral copy numbers of 1.06 × 10^4^ and 7.06 × 10^3^, both of which were statistical different (*P* < 0.05) when compared with PBS control.

## Discussion

Application of shrimp VLP (known to be a dual functioning nano-container for encapsulating several types of cargoes while also serving as an immunostimulant) becomes a well-established platform for halting viral infections in aquaculture [4, 5, 7] Further attempt to gear this MrN-VLP container forward into a versatile delivery system can be done through its chimerization and functional fabrication. Here, we genetically modified MrN-VLP to improve its encapsulation efficacy which eventually enhances a local concentration of therapeutic compound (dsRNA) that acts on the target tissues. One of the pioneering works in container’s interior modification with the aim to enhance its cargo encapsulation is an engineering of the non-viral protein container, lumazine synthase (LS). Mutagenesis of the negatively charged glutamates to replace the exposing luminal foci of LS capsid has greatly enhanced the encapsulation of highly positively charged molecules such as GFP(32+) [23] and deca-arginine enriched HIV protease [24]. Alternatively, modification of this same LS particle by positively charged amino acids could well accommodate with the encapsulation of nucleotide-based cargo [25]. Similar type of modification has also been taken place with VLPs (such as CCMV and MS1 phage) where their cargo enrichment is ultimately aimed [15, 26]. It is anticipated that an enhancement of cargo encapsulation would likely be dependent on electrostatic interaction between capsid luminal surface and loading cargoes.

Structural and sequence analyses reveal that the inner surface of MrNV capsid contains RNA binding domain (RBD) (amino acids 20–29) [16] which is known to play a vital role in the nuclear translocation of MrNV [27]. In addition, the *N*-terminus of MrNV is arginine-rich (highly positive) interacting with either self-borne RNA genome [18] or exogenously synthesized dsRNA [3]. In this study, enhancement of a net-positive charge of MrN-VLP’s luminal surface with 10R peptide (V2 MrN-VLP) consequently improved dsRNA encapsulation efficiency by > 1.4 fold than that of a parental V0-MrN-VLP (Fig. 3). Possession of arginine residues as a domain or cluster in the protein structure has been known to impart many biological functions [28, 29] through an improvement of protein-protein binding, simply through several ionic interactions [30–32]. It should be noted that the replacement of capsid interior with RBD (V1-MrN-VLP) did not increase any encapsulation efficiency beyond deca-arginine peptide – no significant difference was noted between V1- and V2-MrN-VLP, favoring an importance of an electrostatic interaction between capsid and cargo.

To fight against viral infection in shrimp using RNA interference methodology, our first attempt was an encapsulation of VP28 dsRNA into MrN-VLP to alleviate shrimp resistance to WSSV which found to be better than naked dsRNA administration [3, 6]. Besides VP28, VP37, a viral envelop protein involving in the attachment of WSSV virions to the shrimp cells, has also been chosen as a strategic prevention to fight against WSSV viral infection [33, 34]. Inhibition of this viral protein to its receptor using sulfated galactan resulted in weak binding of virus to haemocytes [33]. In addition, interfering of VP37 gene accompanied with VP28 gene by dsRNA that were encapsulated into IHNN-VLP gave a superior protection of the virus infection in shrimp due to synergistically function in knocking down WSSV capsid genes [5]. Here, through modification of VLP interior to accommodate more VP37-dsRNA encapsulation into VLP’s cavity, the significantly higher VP37 gene silencing and viral elimination could be achieved from V2-MrN-VLP than V0-MrN-VLP. We believe that the level of WSSV viral copy number as well as VP37 capsid gene well corresponded with the amount of VP37 dsRNA that was able to load into each type of VLP – the higher loading of dsRNA into V1- and V2-MrN-VLPs than V0-MrN-VLP, the lower WSSV copy number seen in the shrimp tissues (i.e., higher protective effect against the virus). This step-up improvement of MrN-VLP cargo loading has paved a new opportunity of utilizing this interiorly modified MrN-VLP in encapsulating other nucleotide-based compounds for the more advanced vector-based therapy or CRISPR-Cas9 gene knocking down therapy to achieve a better outcome in viral attack. Together with the excellent physical properties of MrN-VLP that we have reported earlier [3, 6], we are currently testing a suspension-type and a feed-pellet mix of dsRNA + MrN-VLP at the semi-field and field trials with the hope to use it as a practical weapon to fight against viral infection in aquaculture field.

### Electronic supplementary material

Below is the link to the electronic supplementary material.

Supplementary Material 1

Supplementary Material 2

## Data Availability

The datasets used and/or analyzed during the current study are available from the corresponding author on reasonable request.

## Declarations

All methods are reported in accordance with ARRIVE guidelines for the reporting of animal experiments. The study was carried out in accordance with the guidelines and regulations approved by the Animal Ethics Committee, Walailak University (Protocol No. 020/2019).
