# Supplementary material for: Interior modification of Macrobrachium rosenbergii nodavirus-like particle enhances encapsulation of VP37-dsRNA against shrimp white spot syndrome infection
Source: BMC Vet Res. 2024 Mar 8;20:91. doi: 10.1186/s12917-024-03936-w (PMC10921773; doi:10.1186/s12917-024-03936-w)
Supplement: Supplementary file 1 — Supplementary Material 1 [file 12917_2024_3936_MOESM1_ESM.doc]

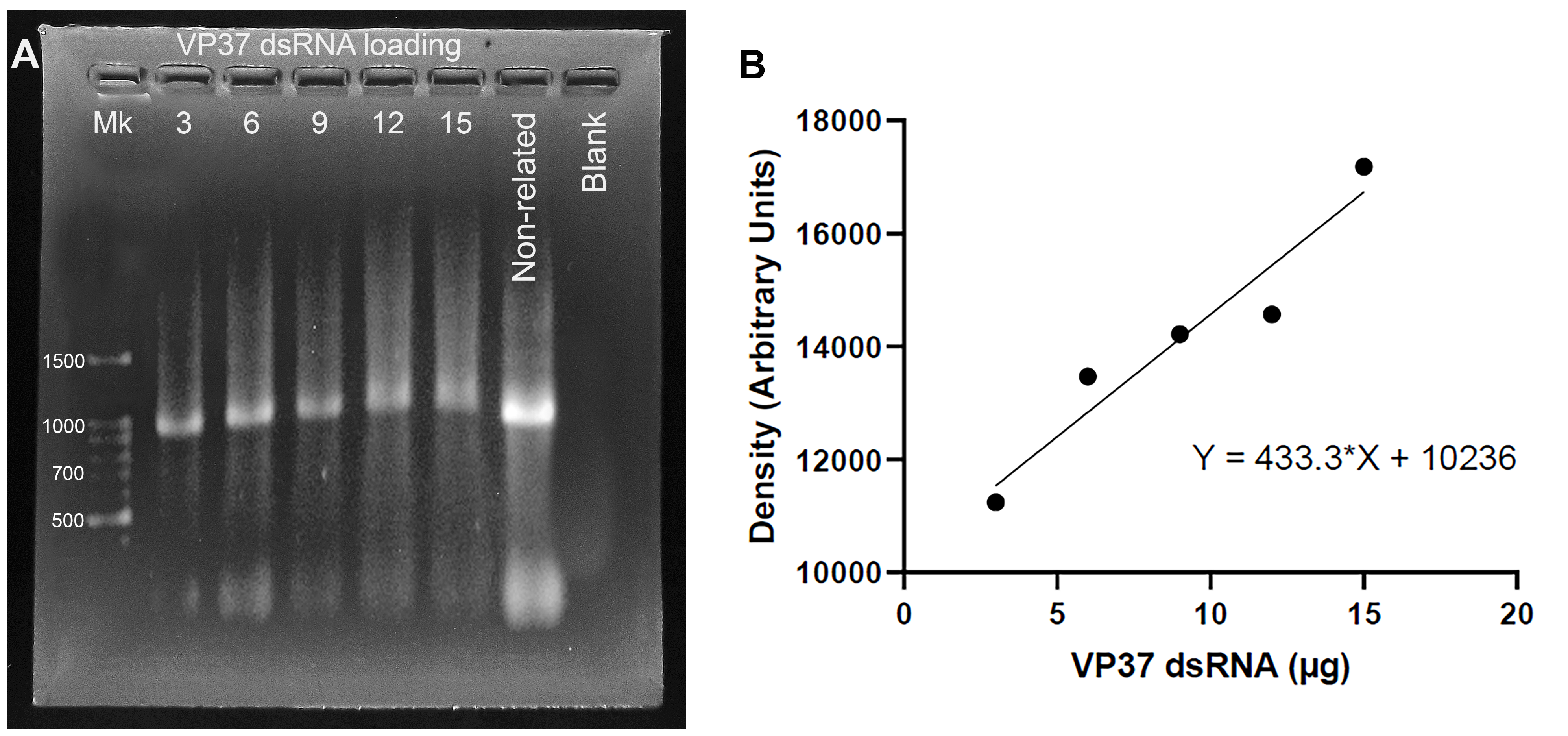


**Supplementary Figure 1** Stand curve of VP37-dsRNA (3-15 ug) resolved in 1% agarose gel electrophoresis (A) and the densitometric analysis of the 843 bp band intensity using an ImageJ software (B).

**Supplementary Table 1** Primers used in this study.

| Genes | Primer sequences |
| --- | --- |
| WSSV(F) | 5′-CCGACGCCAAGGGAACTGT-3′ |
| WSSV(R) | 5′-TTCAGATTCGTTACCGTTTCCA-3′ |
| WSSV_Probe | 5′FAM-CTTCAGCCATGCCAGCCGTCTTCCA-3′BHQ1 |
| WSSV_1s5 (F) | 5′-CTCTGGCAGAATCAGACCAGACCCCTGAC-3′ |
| WSSV_1a16 (R) | 5′-TTCCAGATATCTGGAGAGGAAATTCC-3′ |
| VP37(F) | 5′-GCGGTAAACTTGGATAATGTTCT-3′ |
| VP37 (R) | 5′-TTATGTCCAACAATTTAAAAAGAAGTAGATGA-3′ |
| VP37_T7(F) | 5′-TAATACGACTCACTATAGGGGCGGTAAACTTGG ATAATGTTCT-3′ |
| VP37_T7 (R) | 5′-TAATACGACTCACTATAGGGTTATGTCCAACAA TTTAAAAAGAAGTAGATGA-3′ |
| EF-1α (F) | 5′-GGTGCTGGACAAGCTGAAGGC-3′ |
| EF-1α (R) | 5′-CGTTCCGGTGATCATGTTCTTGATG-3′ |
