# Supplementary material for: Interior modification of Macrobrachium rosenbergii nodavirus-like particle enhances encapsulation of VP37-dsRNA against shrimp white spot syndrome infection
Source: BMC Vet Res. 2024 Mar 8;20:91. doi: 10.1186/s12917-024-03936-w (PMC10921773; doi:10.1186/s12917-024-03936-w)
Supplement: Supplementary file 2 — Supplementary Material 2 [file 12917_2024_3936_MOESM2_ESM.docx]

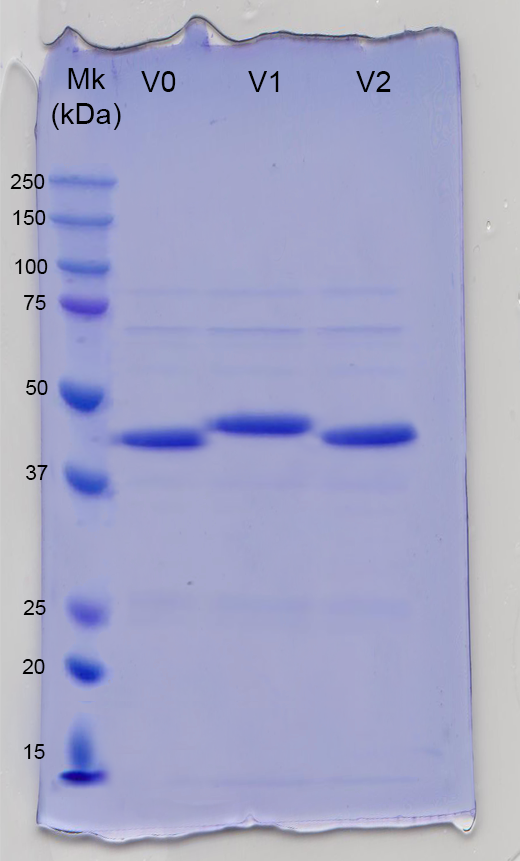


**Figure 2 A** Protein profiles of purified recombinant V0- V1- and V2-MrN-VLPs resolved by 12.5% SDS-PAGE and stained with Coomassie blue.


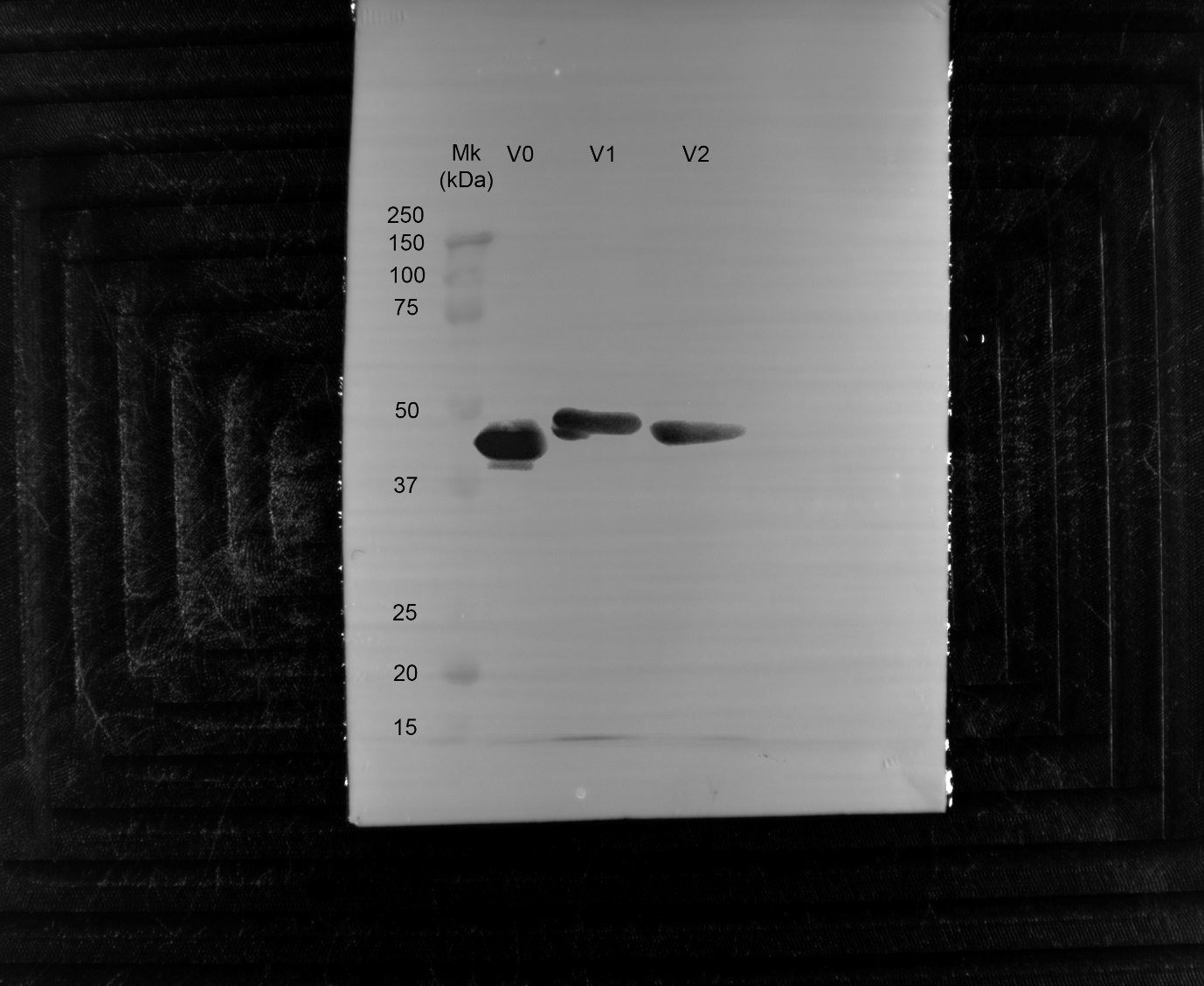


**Figure 2B**  Western blotting of purified recombinant V0- V1- and V2-MrN-VLPs with anti-His antibody. Note a single purified protein band of MrNV capsid protein at 43 kDa.
